# The Availability of Peer Support and Disparities in Outpatient Mental Health Service Use Among Minority Youth with Serious Mental Illness

**DOI:** 10.1007/s10488-020-01073-8

**Published:** 2020-07-29

**Authors:** Victoria D. Ojeda, Michelle R. Munson, Nev Jones, Emily Berliant, Todd P. Gilmer

**Affiliations:** 1 Department of Family Medicine and Public Health, University of California, San Diego, USA; 2 Silver School of Social Work, New York University, New York, NY USA; 3 Department of Psychiatry & Behavioral Neurosciences, Morsani College of Medicine, University of South Florida, Tampa, FL USA

**Keywords:** Minority youth, Mental health service use, Disparities

## Abstract

We examine whether the availability of peer support reduces disparities in service use among minority youth ages 16–24 with serious mental illness in Los Angeles and San Diego Counties. Administrative data from 2015–2018 was used to summarize service use among 13,363 transition age youth age 16–24 with serious mental illness who received services from 183 outpatient public mental health programs; 17.2% were Black, 67.4% were Latinx, and 15.4% were non-Latinx white. The availability of peer support was assessed via a program survey. Generalized linear models were used to assess the relationship between availability of peer support, defined as having a peer specialist on staff, and the annual number of outpatient mental health visits. We also examined the relationship between racial/ethnic concordance of youth and peer specialists and use of outpatient services. Forty-six percent of youth received services from programs that employed peer specialists. Among youth in both counties, the availability of peer support was associated with an increase in annual outpatient visits (P ≤ .05 each). Peer support was associated with reductions in service use disparities among Black and Latinx youth in Los Angeles County (P < .001 each). Peer concordance was associated with an increase in outpatient service use among Latinx youth in both counties (P < .05 each). Peer support was associated with increases in use of outpatient mental health services. Detailed examination of the context for youth peer support implementation is merited to identify the specific pathways that improve outcomes.

Health and social support services provided by peers have the potential to reduce disparities in outpatient service use among minority transition age youth (youth ages 16–24, hereafter youth) with serious mental illness who remain underserved by the public mental health system (Alegria et al. 2016). Reaching and engaging youth with serious mental illness in services is especially important because they often face exceptional challenges during their transition to adulthood, which can result from emancipation from foster care, a lack of natural mentors, and needs that are inadequately met by the mental health system (Cusick et al. 2012; Davis 2003; Munson and McMillen 2009; Vostanis 2005). Compared to youth without serious mental illness, youth with serious mental illness have lower levels of educational attainment, and higher rates of unemployment, poverty, unplanned pregnancy, substance use, homelessness, and justice system involvement (Davis et al. 2004; Davis and Vander Stoep 1997; Vander Stoep et al. 2000; Wagner and Newman 2012). Concerns about the mental health system’s ability to engage and retain youth with serious mental illness in treatment have prompted calls for age-relevant and culturally appropriate services (Edlund et al. 2002; Manteuffel et al. 2008; Pottick et al. 2008).

Minority youth face disparities in both mental health treatment and outcomes, defined as “differences across racial and ethnic groups that are not justified by their underlying health conditions or treatment preferences” (Institute of Medicine 2002; Institute of Medicine and National Research Council 2015). African American and Latinx youth experience higher rates of depression and lower rates of mental health treatment compared to non-Latinx white youth (Alegria et al. 2011; Alegría et al. 2008; Horvitz-Lennon et al. 2009). Disparities in access to care and quality of care persist at the population, provider, and system levels (Cook and Alegria 2011; Cook et al. 2013). These disparities have motivated recommendations for evidence-based, culturally-tailored interventions that emphasize the natural strengths of youth and effectively link them to both professional services and community resources, with the goals of improving access to treatment and promoting equitable health and social outcomes (Alegria et al. 2016).

Peer support services have the potential both to support youth engagement in general and reduce racial/ethnic disparities by providing culturally and developmentally appropriate supports that build on mutuality, empathy, and trust (Grant et al. 2018; Simmons et al. 2017; Solomon 2004). Conceptual frameworks have identified common determinants of engagement among youth, including beliefs about treatment, stigma, and trust (Lal and Malla 2015; Munson et al. 2012; Tindall et al. 2018). Peer support is an intervention that has been shown to have promise for improving mediating factors such as trust and engagement among transition age youth (Gopalan et al. 2017; Munson et al. 2016). Youth peer providers (or peer specialists) have been found to possess unique knowledge and expertise based on lived experience in combination with training that supports skills such as strategic disclosure (Simmons et al. 2020). Peer providers may also bring enhanced credibility and an ability to serve as role models for resilience/recovery (Solomon 2004).

Despite the emergent widespread use of peers in mental health programs for youth, the evidence base for peer support focuses largely on adults and provides mixed results, (Davidson et al. 2012; Lloyd-Evans et al. 2014; Reif et al. 2014), depending on the outcomes assessed (King and Simmons 2018). With respect to reducing disparities, for example, research on adult peer navigation among adults with a range of health conditions, including serious mental illness, seems to support a role of adult peer supporters in increasing engagement and reducing disparities (Corrigan et al. 2017a, b; Corrigan et al. 2014; Corrigan et al. 2017a, b). Overall, these studies suggest good reason to hypothesize that peer support could be effective for reducing disparities among minority youth with serious mental illness, who may benefit from culturally concordant mentorship and support.

In this paper, we examine the availability of peer support among publicly funded outpatient mental health programs in Los Angeles and San Diego Counties. We examine whether programs provide peer support, defined as having a peer specialist on staff. We estimate the effect of having a peer specialist on staff, hypothesizing that youth who receive services from programs that employ peer specialists will have a more favorable service profile, defined as a higher use of outpatient services. We also examine whether disparities in service use exist by race/ethnicity among Black, Latinx, and non-Latinx White youth, and if disparities are less present among programs with peer specialists. Finally, we examine whether peer concordance in terms of race/ethnicity affects service use among minority youth. This paper is part of a larger mixed-methods study that examines the effectiveness of peer support in reducing disparities among minority youth.

## Methods

### Study Sample

Administrative data provided by the Los Angeles County Department of Mental Health and the San Diego County Department of Behavioral Health Services were used to identify Black, Latinx, and White age youth ages 16–24 receiving services premised on a diagnosis of serious mental illness (as defined by the state of California) (2020) who received outpatient mental health services in three fiscal years, from July 1, 2015–June 30, 2018. Data include date of birth, gender, and race and ethnicity; and outpatient service use information including dates of service, diagnosis codes, and procedure codes.

We organized the data by fiscal year. Age was calculated as of January 1 of the fiscal year. Procedure codes identified outpatient services including case management, medication management, rehabilitation, and therapy. We calculated the number of outpatient visits as the number of unique days during which one of these services was received. Diagnoses were examined across the fiscal years and was used to hierarchically assign a diagnosis of schizophrenia, bipolar disorder, or major depression; for example, if a youth received a diagnosis for both schizophrenia and depression, a diagnosis of schizophrenia was assigned.

Youth were assigned to the outpatient program in which they received the highest number of outpatient services during the year; ties were handled by random assignment. The focus of the study was on non-team-based outpatient mental health programs; that is, those providing services such as medication management, counseling, and vocational supports without centralized care coordination. In order to help accurately identify programs serving predominantly youth we limited the sample to sites that served at least 20 youth during any given fiscal year. We excluded programs which reported employment of peer specialists for less than 3 years, in order to ensure that peer specialists could have been accessed by participants throughout the three-year study period.

### Program Survey

We implemented computer-assisted self-administered (CASI) surveys using Qualtrics™, a HIPAA-compliant cloud-based survey software (Provo, UT) to collect program-level data. Contact information was obtained for the programs in which youth were identified as receiving outpatient services. Programs were contacted by phone in order to describe the study and to identify the appropriate persons to respond to the survey. Proposed program responders held a leadership position and had a broad knowledge of the program including the array of services provided and the use of peer specialists.

The program survey was conducted from August 1, 2018 and January 10, 2019. Proposed responders received personalized survey links and non-responders or persons with incomplete surveys received weekly emailed reminders. Responders were also offered the option of completing an interviewer-administered survey on a day/time of their choosing. Electronic informed consent was provided by responders prior to responding to the survey. We sent survey requests to 335 programs; of these, 183 programs (54.6%) responded to the survey, and 76 respondents (45.1%) reported having a peer specialist on staff. About one-third of programs with peer specialists on staff employed peer specialists who were themselves transition aged (16–24; N = 29, 38.2%); 35 programs (46.0%) had peer specialists who were close in age (25–30) and 47 (61.8%) had peer specialist who were over 30 years of age. Among responding programs, 124 (68%) were in Los Angeles County and 59 (32%) were in San Diego County. Among respondents, 157 (85.8%) indicated that they were a program manager, 10 (10.4%) indicated that they were in an executive role, and 7 (3.8%) indicated that they were in a supervisory role.

This study used survey data regarding the array of available services, the availability of peer support services, and whether any peer specialists on staff were Black or Latinx, which was used to calculate the racial/ethnic concordance of youth with peer specialists.

### Analysis Methods

Regression analysis was used to estimate the relationship between outpatient mental health visits and the availability of peer specialist services, adjusting for age, gender, race/ethnicity and clinical diagnosis of the patient, and the array of services available from the outpatient program. The annual number of outpatient mental health services was estimated using generalized linear models assuming a gamma distribution and a log link function (Blough et al. 1999; Manning and Mullahy 2001). Standardized estimates of annual outpatient visits were calculated using recycled predictions (Basu and Rathouz 2005).

In a second set of analyses, we examined the relationship between disparities in service use and the availability of peer specialists. Separate analysis were conducted for youth in programs with and without peer specialists. As described above, standardized estimates were calculated for annual outpatient visits among youth in programs with and without peer specialists for each racial/ethnic group.

In a third set of analyses, we examined the relationship between service use and racial/ethnic concordance between youth clients and peer specialists. These analyses were limited to youth in programs with peer specialists, and separate analyses were conducted for Black and Latinx youth and by county. A variable was created to indicate concordance between the race/ethnicity of the youth client and at least one peer specialist. For example, for Black youth, concordance was assumed if their program reported having a Black peer specialist on staff. Standardized estimates of annual outpatient visits were calculated for youth with and without racial/ethnic concordance with the peer specialists in their programs.

Standard errors for each of the estimates were calculated using the nonparametric bootstrap with clustering at the program level, and p-values were computed using the percentile method from the empirical distributions of the results from 1000 replicates (Efron 1993). This study was approved by the Human Subjects Research Protections Program at the University of California, San Diego, Los Angeles Department of Mental Health, and San Diego County Department of Behavioral Health Services in accordance with the Privacy Rule of the Health Insurance Portability and Accountability Act of 1996.

## Results

### Study Sample

Table 1 shows the characteristics of 13,363 age youth age 16–24 living with serious mental illness who received services from 183 outpatient mental health programs in Los Angeles (N = 9050, 67.7%) and San Diego (N = 4313, 32.3%) Counties. The mean age was 19.1 (SD = 2.7); 7422 (55.5%) were female; 2303 (17.2%) were Black, 9002 (67.4%) were Latinx, and 2058 (15.4%) were non-Latinx White; 2011 (15.1%) received a diagnosis of schizophrenia, 3009 (22.5%) bipolar disorder, and 8343 (62.4%) major depression. Outpatient programs provided a range of services in addition to behavioral health services: most common were family services including family therapy and parenting classes, employment support, and housing support. There were 6194 (46.4%) youth in programs with peer specialists on staff, and 7169 (53.7%) in programs without peer specialists.

**Table 1:** Characteristics of transition age youth receiving services in Los Angeles and San Diego counties

	Overall		Los Angeles county		San Diego county		P value
	N	%	N	%	N	%	
Overall	13,363		9044		2173		
Age group							< .001
Age 16–17	5698	42.6	3663	40.5	2035	47.2	
Age 18–19	2760	20.7	1996	22.1	764	17.8	
Age 20–21	1855	13.9	1312	14.5	543	12.6	
Age 22–23	1835	13.7	1250	13.8	585	13.6	
Age 24	1215	9.1	829	9.2	386	9.0	
Gender							.002
Female	7422	55.5	5111	56.5	2311	53.6	
Male	5941	44.5	3939	43.5	2002	46.4	
Race/ethnicity							< .001
Black	2303	17.2	1776	19.6	527	12.2	
Latinx	9002	67.4	6424	71.0	2576	59.8	
Non-Latinx White	2058	15.4	850	9.4	1208	28.0	
Diagnosis							< .001
Schizophrenia	2011	15.1	1108	12.2	903	20.9	
Bipolar disorder	3009	22.5	1789	19.8	1220	28.3	
Major depression	8343	62.4	6153	68.0	2190	50.8	
Service array: services available in addition to behavioral health							
Basic services (e.g. ID, transportation, laundry)	4400	32.9	3352	37.0	1048	24.3	< .001
Benefits management	1738	13.0	1157	12.8	581	13.5	.270
Education Support	5930	44.4	4124	45.6	1806	41.9	< .001
Employment support	7505	56.2	5600	61.9	1907	44.2	< .001
Family services (e.g. family therapy, parenting skills)	8513	63.7	5990	66.2	2523	58.5	< .001
Financial skills	4885	36.6	3675	40.6	1210	28.1	< .001
Housing support	6980	52.2	5085	56.2	1895	43.9	< .001
Physical health services	2507	18.8	2046	22.6	461	10.7	< .001
Enrollment in a program with peer specialists							< .001
Program has peer specialists on staff	6194	46.4	3856	42.6	2338	54.2	
Program does not have a peer specialist on staff	7169	53.7	5194	57.4	1875	45.8	

There were significant differences among youth in our sample based on the two counties where youth were served (Table 1). Youth served in Los Angeles County were somewhat older, more likely to be female, and more likely to be Black or Latinx, but were less likely to be diagnosed with schizophrenia or bipolar disorder than youth who received services in San Diego County (P ≤ 0.002 each). Outpatient programs in Los Angeles County were generally more likely to provide additional types of services beyond behavioral health services. Programs providing services to youth in Los Angeles County were also less likely to have a peer specialist on staff when compared to programs in San Diego County (P < 0.001). Given these significant differences in youths’ and programs’ characteristics, we decided to analyze the data separately by county.

### Peer Specialists and Service Use

Table 2 shows standardized estimates of number of annual outpatient visits among youth in programs with and without peer specialists stratified by county. Overall, Los Angeles County exhibited a greater mean number of annual outpatient visits compared to San Diego County (P < 0.001). In both counties, youth in programs with peer specialists on staff had a greater number of annual outpatient visits than youth in programs without peer specialists: 17.7 (SE = 0.4) vs. 16.7 (SE = 0.6) in Los Angeles County (P = 0.031) and 13.0 (SE = 0.4) vs. 10.9 (SE = 0.3) in San Diego County (P < 0.001), representing increases of 6% and 19%, respectively.

**Table 2:** Standardized estimates of numbers of mental health outpatient visits among transition age youth in programs with and without peer specialists

County	Mental health outpatient visits in programs with peers		Mental health outpatient visits in programs without peers		Difference in mental health outpatient visits		P value
	Mean visits	SE	Mean visits	SE	Mean visits	SE	
Los Angeles county	17.7	.4	16.7	.3	1.0	.6	.031
San Diego county	13.0	.4	10.9	.3	2.1	.6	< .001

### Peer Specialists and Racial/Ethnic Disparities in Service Use

Table 3 examines the relationship between having a peer specialist and racial and ethnic disparities in outpatient service use. There were significantly greater annual outpatient mental health visits among Black and Latinx youth in programs with peer specialists in Los Angeles County, compared to Black and Latinx youth in programs without peer specialists: 5.4 (SE = 1.4) and 2.9 (SE = 1.0) greater visits, respectively (P < 0.001 each). In San Diego County, Latinx youth in programs with peer specialists had a 1.4 (SE = 0.9) greater outpatient visits compared to Latinx youth in programs without peer specialists (P = 0.043).

**Table 3:** Standardized estimates of numbers of mental health outpatient visits among transition age youth in programs with and without peer specialists

County	Mental health outpatient visits in programs with peers		Mental health outpatient visits in programs without peers		Difference in mental health outpatient visits		P value
	Mean Visits	SE	Mean Visits	SE	Mean Visits	SE	
Los Angeles county							
Black	20.8	1.1	15.4	.8	5.4	1.4	< .001
Latinx	19.7	.9	16.8	.4	2.9	1.0	< .001
Non-Latinx White	19.6	1.3	19.3	1.0	.3	1.6	.398
San Diego county							
Black	11.9	.8	12.4	1.0	-.6	1.4	.666
Latinx	12.4	.7	11.0	.6	1.4	.9	.043
Non-Latinx White	12.0	.7	12.0	.7	-.1	1.0	.505

In order to evaluate disparities in service use, we examined whether outpatient service use was lower among Black or Latinx youth compared to non-Latinx white youth, in programs with and without peer specialists, in Los Angeles and San Diego Counties. In Los Angeles County, outpatient mental health service use was significantly lower among both Black and Latinx youth in programs without peer specialists, compared to non-Latinx White youth (P < 0.001 each); these disparities did not exist in programs with peer specialists. There were no significant disparities in outpatient services use by race/ethnicity among programs with or without peer specialists in San Diego County.

### Racial/Ethnic Concordance with Peer Specialists

Table 4 shows racial and ethnic concordance between youth and peer specialists among programs with a peer specialist on staff. Concordance ranged from 31.0% among Black youth in San Diego County to 90.4% among Latinx youth in Los Angeles County.

**Table 4:** Racial/ethnic concordance among transition age youth and peer specialists

County	Overall		Youth in concordance with peer specialist	
	N	%	N	% concordance
Los Angeles county				
Black	815	23.5	569	69.8
Latinx	2655	76.5	2400	90.4
San Diego county				
Black	332	21.1	103	31.0
Latinx	1241	78.9	773	62.3

Table 5 shows the relationship between racial and ethnic concordance and outpatient service use. There were no significant differences related to concordance among Black youth in either county. However, in both counties, Latinx youth in programs with concordance with peer specialists had a greater number of annual outpatient mental health visits: 3.0 (SE = 1.2, P = 0.014) and 6.4 (SE = 1.1, P < 0.001) greater visits in Los Angeles and San Diego Counties, respectively.

**Table 5:** Standardized effects of concordance among transition age youth and peer specialists

County	Outpatient programs with concordant peer specialists		Outpatient programs with non-concordant peer specialists		Difference in mental health outpatient visits		P value
	Mean visits	SE	Mean visits	SE	Mean visits	SE	
Los Angeles county							
Black	20.7	1.6	19.2	2.0	1.5	2.6	.218
Latinx	15.5	.6	12.5	1.0	3.0	1.2	.014
San Diego county							
Black	13.8	1.8	11.1	.9	2.7	2.1	.112
Latinx	14.9	.7	8.5	.6	6.4	1.1	< .001

## Conclusions

In this paper, we examined the relationship between availability of peer support and the use of outpatient mental health services among minority youth with serious mental illness in Los Angeles and San Diego Counties. We found that having a peer specialist on staff was associated with increases in outpatient service use in both counties, and with reduced disparities in service use among Black and Latinx youth in Los Angeles County. The availability of a peer specialist with racial/ethnic concordance was also associated with greater outpatient service use among Latinx youth in both counties. These results suggest that peer support services are a promising approach to reducing the documented low rate of continued engagement in mental health services among youth (Pottick et al. 2014; Ringeisen et al. 2016).

Studies conducted with adults living with serious mental illness generally find that peers are effective in supporting client use of mental services (Davidson et al. 2012), as was true within our study. Factors that may contribute to this outcome include a ‘do whatever it takes’ approach including highly flexible positions, modeling of service use, and provision of instrumental, informational, and emotional support (Simmons et al. 2017; Watson 2019). Other adult-based studies find that peers provide hope for a trajectory of recovery, which may be particularly important for youth (Repper and Carter 2011; Walker and Bryant 2013). Peer providers may also contribute to or strengthen programs’ recovery orientation (Jones et al. 2020; Thomas and Salzer 2018). Given the massive growth in the peer workforce over the past decade, including youth-focused peer certification standards (Myrick and Del Vecchio 2016; Wolf 2018), it is critical that the field directly investigate youth peer support implementation, roles, benefits and underlying processes, while drawing on but not assuming the generalizability of adult peer support studies.

Our findings suggest that the availability of peer staff within youth mental health programs may contribute to greater outpatient service utilization among minority youth and that racial/ethnic concordance may further support service uptake among Latinx youth. Prior studies have found mixed evidence for the role of provider–client concordance, though fewer studies on this topic have been conducted with youth living with mental illness (Cabral and Smith 2011; Valenzuela and Smith 2016). Hiring of minority peer specialists may reflect greater cultural sensitivity/competency within the program, and/or minority peer staff may contribute to a culturally sensitive climate (Jones et al. 2020). Racial/ethnic concordance between peers and youth may play a positive role by increasing client trust and rapport (Corrigan et al. 2018; Corrigan et al. 2017a, b), or by helping youth manage family and/or community stigma regarding mental illness (Greden et al. 2010; Rivera-Segarra et al. 2019). These possible interpretations all merit further exploration, including qualitative interviews designed to help unpack underlying directionality, processes and mechanisms. In addition to race/ethnicity, research into the age and sexual minority group identification of peer providers, and their contribution not just to a recovery orientation but also a youth orientation may prove key to understanding unique impacts within this population.

Our findings regarding racial/ethnic concordance also raise important, unanswered questions regarding the role of “shared experience.” To our knowledge, only one study has actually probed what it is that service users perceive to be the most important aspect of shared experience. In this provocative study, researchers found that Veterans with mental health challenges rated shared military experience and trauma as more significant than their shared experience receiving mental health or substance use services (Clark et al. 2016). While mental health is often assumed to be the defining aspect of shared experience in the context of peer support, no empirical evidence directly attests to this, and the above work raises questions about what youth prioritize in their relationships with peer providers. These questions might also be pursued with respect to shared experience of particular kinds of systems: for example, having (versus not having) shared experience with child welfare or juvenile justice.

Our differential findings by county speak to the importance of context and the importance of examining interventions, including peer support, by county or administrative catchment area. These data can also inform future research focused on specific adaptations based on context and other factors, such as youth preferences. Future research can examine different types of peer support and what it is about the support that is vital for youth, which may vary by county. Our sample of youth in Los Angeles County were largely minority youth and they had higher rates of concordance with peer specialists. Youth in San Diego County were younger and had higher rates of more serious diagnoses including schizophrenia and bipolar disorder. These youth also had greater access to peer specialists.

This paper had a few limitations. Our analyses focused on commonly used mental health services that were captured in administrative data. We may have missed changes in other types of services that were not captured in the data such as non-clinical, psychosocial interventions. Peer services may span many different types of interactions/engagements, and availability of peer support may affect some of these more than others. We did not have data on mental health outcomes or functioning, or outcomes such as resilience and self-efficacy that are often a focus of peer support. We did not have data on the numbers of peer specialists or their hours worked. We measured peer support using a point in time survey of program managers, and our study design compared programs that have a peer specialist on staff to those that did not; although we were able to control for a range of services provided by the programs, it is possible that programs that had a peer specialist on staff were different from programs that do not employ peer specialists in ways that we did not measure yet could affect outcomes.

Despite these limitations, the present study provides useful information on a large sample of youth with serious mental illness. Our data show that having a peer specialist on staff within a youth program is associated with increased use of outpatient mental health services within that program. Future research should consider the specific roles and characteristics of peer specialists working in youth settings, as well as the context in which services are delivered and the considerations that affect whether outpatient programs provide peer support.
